# Efficient Editing of the Nuclear *APT* Reporter Gene in *Chlamydomonas reinhardtii* via Expression of a CRISPR-Cas9 Module

**DOI:** 10.3390/ijms20051247

**Published:** 2019-03-12

**Authors:** Daniel Guzmán-Zapata, José M. Sandoval-Vargas, Karla S. Macedo-Osorio, Edgar Salgado-Manjarrez, José L. Castrejón-Flores, María del Carmen Oliver-Salvador, Noé V. Durán-Figueroa, Fabien Nogué, Jesús A. Badillo-Corona

**Affiliations:** 1 Instituto Politécnico Nacional, Unidad Profesional Interdisciplinaria de Biotecnología. Av. Acueducto S/N., Col. Barrio La Laguna Ticomán, 07340 Mexico City, Mexico; danielguza@gmail.com (D.G.-Z.); ibt_jose_sandoval@hotmail.com (J.M.S.-V.); karla_032214@hotmail.com (K.S.M.-O.); esalgado@ipn.mx (E.S.-M.); jlcastrejon@ipn.mx (J.L.C.-F.); moliver@ipn.mx (M.d.C.O.-S.); nduranf@ipn.mx (N.V.D.-F.); 2 Institut Jean-Pierre Bourgin, INRA, AgroParisTech, CNRS, Université Paris-Saclay, 78000 Versailles, France

**Keywords:** CRISPR/Cas9, *Chlamydomonas reinhardtii*, adenine phosphoribosyl transferase, APT, Cas9, glass beads, particle bombardment

## Abstract

The clustered regularly interspaced short palindromic repeat/CRISPR-associated protein 9 (CRISPR/Cas9) technology is a versatile and useful tool to perform genome editing in different organisms ranging from bacteria and yeast to plants and mammalian cells. For a couple of years, it was believed that the system was inefficient and toxic in the alga *Chlamydomonas reinhardtii*. However, recently the system has been successfully implemented in this model organism, albeit relying mostly on the electroporation of ribonucleoproteins (RNPs) into cell wall deficient strains. This requires a constant source of RNPs and limits the application of the technology to strains that are not necessarily the most relevant from a biotechnological point of view. Here, we show that transient expression of the *Streptococcus pyogenes* Cas9 gene and sgRNAs, targeted to the single-copy nuclear *apt9* gene, encoding an adenine phosphoribosyl transferase (*APT*), results in efficient disruption at the expected locus. Introduction of *indels* to the *apt9* locus results in cell insensitivity to the otherwise toxic compound 2-fluoroadenine (2-FA). We have used agitation with glass beads and particle bombardment to introduce the plasmids carrying the coding sequences for Cas9 and the sgRNAs in a cell-walled strain of *C. reinhardtii* (CC-125). Using sgRNAs targeting exons 1 and 3 of *apt9*, we obtained disruption efficiencies of 3 and 30% on preselected 2-FA resistant colonies, respectively. Our results show that transient expression of Cas9 and a sgRNA can be used for editing of the nuclear genome inexpensively and at high efficiency. Targeting of the *APT* gene could potentially be used as a pre-selection marker for multiplexed editing or disruption of genes of interest.

## 1. Introduction

Since its development as a biotechnological tool and its first applications, CRISPR/Cas9 technology has been proposed as a simple and precise tool for genome editing [1,2,3]. It has been successfully established in organisms across all kingdoms of life, including bacteria [4], yeasts [5], plants [6], mice [7] and human cells [3,8]. The technology is simple, yet fascinating and precise, requiring only three basic components: the nuclease CRISPR-associated protein 9 (Cas9), a CRISPR RNA (crRNA), containing a 20-nt sequence complementary to the target DNA, and a *trans*-activating crRNA (tracrRNA), which functions as a recognition scaffold structure for Cas9. The last two components, crRNA and tracrRNA, can be physically linked to form what is called the single guide RNA (sgRNA) [2]. In its most basic application, genome editing is achieved by constitutive or transient expression from vectors containing the Cas9 and sgRNA sequences in the cells of interest. Alternatively, a pre-assembled Cas9-sgRNA ribonucleoprotein (RNP) can be directly delivered into the cells by electroporation [9,10,11,12]. Following expression, or introduction, of the Cas9-sgRNA complex, recognition of the target sequence results in a double-stranded cleavage 3–4 nt upstream of the protospacer adjacent motif (PAM) [13,14]. Double-stranded cleavages can then be repaired by non-homologous end joining (NHEJ), resulting in random insertions and deletions (*indels*), or by homologous recombination (HR) using double- or single-stranded DNA donors, resulting in a finer, more controlled editing of the target site [10]. Furthermore, engineered forms of Cas9 and algorithm-mediated selection of non-redundant target sites make the technology more versatile, precise and reproducible than previously developed methods for genome editing, such as meganucleases, zinc fingers, and TALENs [15].

The green unicellular alga *Chlamydomonas reinhardtii* is a widely used model organism for the study of photosynthesis, flagellum structure and function, chloroplast biogenesis and phototaxis [16]. This eukaryotic organism is considered to have a great potential in biotechnology for the production of value-added molecules [17] and has recently been proposed as a sustainable photosynthetic chassis for synthetic biology [18]. However, where other organisms (e.g., *Escherichia coli* and *Saccharomyces cerevisiae*) have benefited from targeted inactivation of genes using tools that take advantage of homologous recombination (HR), *Chlamydomonas* has suffered the consequences of the low frequency at which this event occurs in the nuclear genome. A recent effort has generated a library of insertional mutants in which 1562 (9%) of the 17,737 predicted genes (genome assembly v5.3) have been disrupted [19]. Even though this library represents a good starting point for carrying out a more systematic approach to elucidate gene function in a genome wide scale, a reliable tool allowing precise targeting of nuclear genes would greatly benefit the research focused on this organism around the world. Such a tool could give a boost to applications ranging from forward and reverse genetics to metabolic engineering and top-down synthetic biology, making scientific progress advance at an accelerated pace.

Implementation of CRISPR/Cas9 in *Chlamydomonas* has not been as straightforward and as efficient as in other model organisms. The initial implementation yielded only one colony with a modified target locus per 1.5 × 10^9^ initial cells used for transformation. This extremely low efficiency was attributed to Cas9 being potentially toxic to the cells [20]. This somewhat discouraging result was followed by the successful electroporation of pre-assembled Cas9/sgRNA RNP complexes into cells resulting in the edition of the photosynthesis-related genes *CpSRP43*, *ChlM*, *ZEP* and *CpFTSY* [9,12]. Targeting of *CpSRP43*, and *ChlM* genes had the advantage of allowing pre-selection of cells, as they showed a distinctively light green to yellow phenotype, indicating a potentially edited locus. In this case, the editing/disruption efficiency was in the range of 0.17–1.4%, calculated as the ratio of confirmed editions/pre-selected colonies with a green or yellow phenotype [12]. In the case of *ZEP* and *CpFTSY*, the edition efficiency was in the range of 0.007–0.56% [9], as measured by targeted deep sequencing of total genomic DNA extracted from the entire transformed culture. In both cases, the edition/disruption efficiency was very low (approximately 1 edition per 10^7^ initial cells), but the results were encouraging this time, as they showed that the supposed toxicity of Cas9 could be overcome with the use of RNP, albeit at the cost, both monetary and in terms of labor, of pre-assembling the Cas9/sgRNA complexes [9,12]. A year later, again using an approach that consisted of using genetically encoded Cas9 and sgRNA, Jiang et al. [21] demonstrated that by transiently expressing a gene-within-a-gene Cas9/sgRNA hybrid, repairing by edition of a bleomycin resistance-conferring gene and rescue of arginine auxotrophy, by editing the *ARG* gene in the auxotrophic mutant (*arg7*), could be achieved at a frequency of 1 successfully edited colony per 2.5–3.3 × 10^7^ initial cells. Recently, Greiner et al. [10] showed that a higher editing efficiency is possible. Disruption of the phytoene synthase-1 gene (*PSY1*), which produces white colonies that are easy to identify, resulted in an edition efficiency of up to 9% in preselected white colonies when using a genetically encoded Cas9 from *Staphylococcus aureus.* A lower efficiency, up to 3.3%, was obtained with the Cas9 from *S. pyogenes.* In both cases, a cell-wall deficient strain (CC-3403) was used for electroporation-mediated introduction of the transformation plasmids. Similarly, four photoreceptor genes were targeted for disruption: the *ChR2* (*ChR2-sg*), chlamyopsin-1/2 (*COP1/2*-*sg*), chlamyopsin-5 (*COP5*-*sg*), and phototropin (*PHOT*-*sg*) genes. Greiner et al. [10] have also optimized transformation with preassembled Cas9/gRNA RNP complexes. Using different strains (CC-3403, CC-125, SAG73.72, and 3403-*uvr8*-*2*), a total of eight different genes (*COP1/2*, *COP5*, *aCRY*, *PHOT*, *UVR8*, *VGCC*, *MAT3*, *KU80*, and *POLQ*) were successfully disrupted using various homology-directed repair donors and three marker genes. More recently, the use of the Cas9 ortholog ribonucleoprotein Centromere and Promoter Factor 1 (Cpf1) has been shown to be more efficient at homology-directed DNA replacement through the use of single-stranded oligodeoxynucleotides as repair templates in *C. reinhardtii* [22].

As we have described, the methods with the highest disruption efficiency for nuclear genes in *Chlamydomonas*, in which a genetically encoded CRISPR/Cas9 system has been delivered to the cells, rely on the selection of stably transformed lines in antibiotic-containing media. However, constitutive expression of Cas9, the sgRNA and the selectable marker is not needed once disruption/edition of the genes of interest has been accomplished, and could potentially be a risk for disruption of off-target genes. Here, we wanted to examine if transient expression of *S. pyogenes* Cas9 and sgRNAs, encoded in two independent plasmids, could be used for edition/disruption of nuclear genes in *C. reinhardtii*. We show that, following transformation using glass beads and particle bombardment, transient expression of *S. pyogenes* Cas9 and its cognate sgRNA, targeting the nuclear *APT,* results in disruption of this gene and yields cells that are resistant to the otherwise toxic compound 2-FA. This method could be useful for efficient knock-out of genes for which disruption does not necessarily lead to a screenable phenotype. Furthermore, the strategy presented here can be applied to cell-walled *C. reinhardtii* strains, thus opening the use of the CRISPR gene editing tool to biotechnological applications.

## 2. Results

To investigate whether transient expression of *S. pyogenes* Cas9 along with its cognate sgRNA could be used for genome editing in the photosynthetic alga *C. reinhardtii*, we chose to target the Adenine phosphoribosyl transferase (*APT*) gene. The product of this gene participates in the purines salvage pathway, a mechanism that allows the cells to recover part of the free nucleic acids that result from degradation processes or cell death [23]. The APT enzyme catalyzes the conversion of free adenines into their nucleotide adenosine monophosphate form, but can also use adenine analogues as substrates. These analogues include 2-fluoroadenine (2-FA), 2,6-diaminopurine, and 6-methylpurine, which are toxic to cells when metabolized [24]. To the best of our knowledge, *knocking-out* of the *APT* gene in *C. reinhardtti* using CRISPR/Cas9 has not been attempted. We reasoned that a Cas9-mediated double strand break (DSB) in the coding region of the *APT* gene in *C. reinhardtii* would result in loss of function in this gene, following insertions or deletions (*indels*) caused by the error-prone Non-homologous end joining/Alternative end joining (NHEJ/AltEJ) repair mechanisms [10].

**Figure 1:**
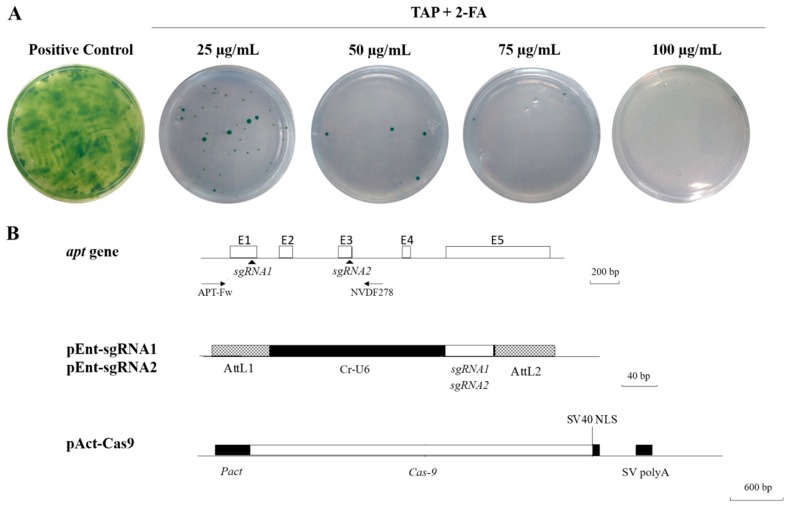
Determination of the minimum inhibitory concentration of 2-FA in the growth of *C. reinhardtii* and transformation vectors. (**A**) Wild-type cells of *C. reinhardtii* CC-125 (2 × 10^8^) were plated on solid TAP media supplemented with different concentrations of 2-FA (25, 50, 75 and 100 μg/mL). Pictures shown correspond to 2-week-old cultures. Experiments were carried out in triplicate. (**B**) Schematic representation of the *apt* gene and vectors carrying the sgRNAs and the Cas9 genes. The *apt* gene shows annealing sites for the sgRNAs and primers used for amplification and sequencing of DNA fragments to verify edition.

To test our hypothesis, we first determined the minimum inhibitory concentration of 2-FA on the growth of *Chlamydomonas* in solid media. We plated 2 × 10^8^ wild-type *C. reinhardtii* CC-125 (mt+) cells on solid TAP media supplemented with different concentrations of 2-FA (25, 50, 75 and 100 μg/mL). After two weeks of incubation, we observed scattered growth of colonies in a proportionally inverse relation in plates containing 25–75 μg/mL of 2-FA (Figure 1A). Only in the plates with a concentration of 100 μg/mL 2-FA we consistently observed inhibition of cell growth. We decided to use this concentration, 100 μg/mL 2-FA in TAP media, to carry out the experiments described below.

To disrupt the *C. reinhardtii APT* gene (NCBI; Gene ID: 5717232), we designed two sgRNAs to target exons 1 (sgRNA1) and 3 (sgRNA2) of the 5 exons that the gene contains. The two sgRNAs, present in vectors pEnt-sgRNA1 and pEnt-sgRNA2, were expressed from a *C. reinhardtii* U6 promoter. The *S. pyogenes* Cas9, contained in vector pAct-Cas9, was expressed from the rice actin promoter and the NOS terminator (Figure 1B). Vectors pAct-Cas9 and pEnt-sgRNA carry selectable marker cassettes that confer resistance to kanamycin, but we did not place transformed *C. reinhardtii* cells under selection with this antibiotic. We reasoned that after entering the cells, the genes encoding the Cas9 and sgRNAs would be transiently expressed, thus targeting the *APT* gene, resulting in disruption through *indels*, which would yield cells able to proliferate in the presence of 2-FA. As the plasmids lack a functional replication origin, they would be lost after a few generations.

**Figure 2:**
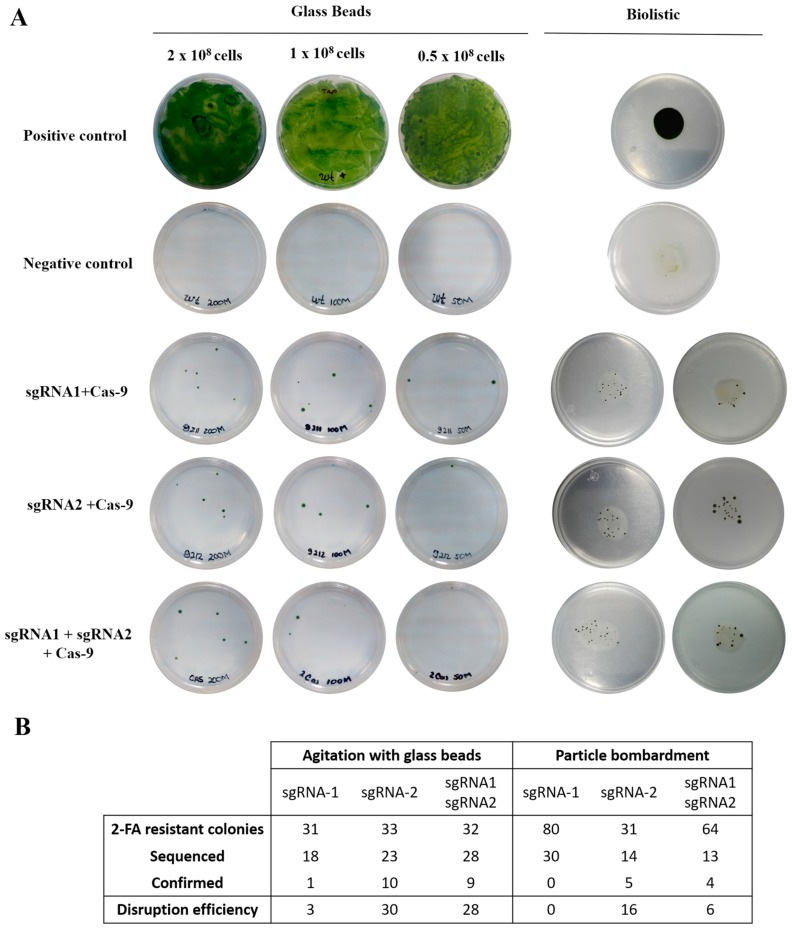
Selection of colonies resistant to 2-FA after transformation and edition efficiency. (**A**) *Chlamydomonas reinhardtii* strains recovered in 2-FA following transformation with different combinations of plasmids pAct-Cas9, pEnt-sgRNA1 and pEnt-sgRNA2. All plates shown contain TAP media supplemented with 100 μg/mL of 2-FA except for the positive control which corresponds to the wild type *C.reinhardtii* strain, plated in solid TAP media without 2-FA. (**B**) Disruption efficiencies as a percentage for the two methods used for transformation. Disruption efficiency was calculated as the ratio of confirmed edited colonies/2-FA resistant colonies and multiplied by 100.

We transformed *C. reinhardtii* cells by the glass beads method [25] with the following combination of vectors: pEnt-sgRNA1+pAct-Cas9, pEnt-sgRNA2+pAct-Cas9 and pEnt-sgRNA1+pEnt-sgRNA2+pAct-Cas9 (Figure 2A). Culture with different cell densities (0.5 × 10^8^, 1 × 10^8^ and 2 × 10^8^ cells) were used for transformation and then incubated in liquid TAP media without antibiotics to allow for cell to recover for 24 h before plating them in TAP media supplemented with 2-FA for selection of transformed cells. Resistant colonies began to appear after 8 days and were completely developed by day 14. After this period, individual colonies were recovered and maintained in solid TAP media supplemented with 2-FA (100 μg/mL). The number of colonies obtained was directly proportional to the initial number of cells, on average we obtained 6, 4 and 2 resistant colonies for 2 × 10^8^, 1 × 10^8^ and 0.5 × 10^8^ initial cells, respectively. Following 3 transformation events, we obtained a total of 31 (mean 10.3, standard deviation 1.5), 33 (mean 10, std. dev 0) and 32 (mean 10.3, std. dev 0.5) resistant colonies to 2-FA for plasmid combinations pEnt-sgRNA1+pAct-Cas9, pEnt-sgRNA2+pAct-Cas9 and pEnt-sgRNA1+pEnt-sgRNA2+pAct-Cas9, respectively (Figure 2B).

**Figure 3:**
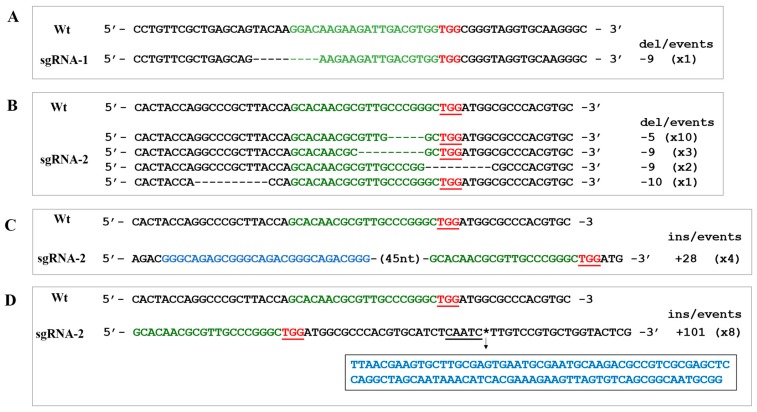
Edition of the *APT* gene in *C. reinhardtii* and analysis of the *indels* generated. (**A**) Deletion of 9 nt observ ed after a canonical cleavage with the sgRNA-1. (**B**) Deletions observed after canonical cleavage with the sgRNA-2. (**C**) Insertions observed upstream of the PAM site after non-canonical cleavage with sgRNA-2. (**D**) Insertions observed downstream of the PAM site with sgRNA-2. PAM sites are indicated in red and underlined. Del: deletions, indicated with a (−) sign; ins: insertions, indicated with a (+) sign. Number in parenthesis indicate the number of observed colonies with that particular *in*/*del*.

We then sequenced a fragment of the *APT* gene in the resistant colonies obtained. Using DNA extracted from all 2-FA resistant colonies and primers that span the targeted loci (APTFw and NVDF278; Figure 1B and Supplemental Figure S1), a PCR was performed to amplify this region. In more than 50% of the colonies (18, 23 and 28; Figure 2B), we were able to amplify a fragment of roughly the expected size (~1131 bp, Supplemental Figure S2). Failure to amplify a PCR product in about 50% of the colonies could be the result of large insertions or deletions taking place in the DSB generated by Cas9. An atypical gene modification has already been observed in *Chlamydomonas*, where apparently, large insertions seem to be predominant over small indels [9]. A similar result was also observed by Greiner et al. [10]; it is likely that large insertions are taking place in the lines where we failed to obtain a PCR product; however, we did not follow-up on these potentially large insertions. We purified the ~1.1 kb PCR products and had them sequenced (Supplementary Data 1). Although the number of 2-FA resistant colonies was fairly similar for transformation with sgRNA1, sgRNA2 and sgRNA1+sgRNA2 the efficiency of *APT* disruption differed notably. With the sgRNA1, targeting exon 1, disruption of the *APT* gene was confirmed in only 1 colony, which represents a 3% disruption efficiency (calculated as the ratio of 2-FA resistant colonies/confirmed disruption by sequencing). Analysis of the *APT* sequence in this line revealed a 9-nt deletion located in part of the sgRNA1 target but at a distance of 16-nt from the PAM site (Figure 3A), not fitting the canonical cleavage distance, ~3–4 bp upstream of the PAM sequence [26], but reminiscent of what has already been observed in animal cells [27,28]. By contrast, when sgRNA2, targeting exon 3, and sgRNA1/sgRNA2 were used, disruption of the *APT* gene was confirmed in 10 and 9 colonies, respectively. These represent a disruption efficiency on preselected 2-FA resistant colonies of 30 and 28%, respectively. Analysis of the 19 sequences where the *APT* gene was disrupted (10 from colonies obtained after transformation with sgRNA2 and 9 from colonies obtained after transformation with sgRNA1/sgRNA2), showed that most DSB occurred two nucleotides upstream of the PAM site (Figure 3B), although there were DBS breaks that occurred up to 23 nucleotides upstream of the PAM site (not shown), similar to the result obtained with sgRNA1. Error-prone NHEJ/AltJ resulted in deletion of 5–10 nucleotides. Analysis of the 9 events obtained combining the two sgRNAs (sgRNA1/sgRNA2) showed that disruption of the *APT* gene occurred exclusively in exon 3, reinforcing the previous observation that sgRNA1 has a lower efficiency than sgRNA2. We attribute this difference in the efficiency to CG content and the presence of a guanine proximal to the PAM site, a characteristic that has been reported before [29]. When both sgRNAs were analyzed with respect to secondary structures using the RNAfold web server (http://rna.tbi.univie.ac.at/cgi-bin/RNAWebSuite/RNAfold.cgi; Supplemental Figure S3), sgRNA2 showed a more accurate functional predicted folding than sgRNA1 [30]. However, as reported by Thyme et al. [31], these observations are not general rules and may depend on the organism and the nuclease worked with, and this will need further study to improve successful editing in *C. reinhardtii*. 

We carried out similar transformations with particle bombardment and recovered a greater number of colonies resistant to 2-FA, a total of 175 2-FA resistant colonies from two transformation events were obtained (Figure 2A,B). From these, we sequenced 30, 14, 13 PCR products obtained from colonies obtained with sgRNA1, sgRNA2 and sgRNA1/sgRNA2, respectively. In this case, we were able to confirm disruption of the *APT* gene in 5 of the lines obtained with sgRNA2 and in 4 of the lines obtained with sgRNA1/sgRNA2, representing a 16 and 6% disruption efficiency, respectively (Figure 2B). Interestingly, in 8 out of these 9 lines, we found a fragment of 101-bp 24 nucleotides downstream of the PAM site (Figure 3D). We carried out a BLAST search and identified this sequence as a fragment of the MRC1 (Mrc1) miniature retrotransposon [32]. Mrc1 is a retrotransposon element of 1625 bp consisting on ~420-bp unique sequence (from 604–1022) bracketed by ~600-bp long terminal repeats (LTRs: 1–603 and 1023–1625), the insertion of fragments from this element are presumably mediated by 5–7-bp duplications in target sites. This 5–7 bp sequence is AAGATTG, which is present at 2 loci in the *APT* gene, one of them in the middle of sgRNA1 (position 263 of the *APT*) and the other one exactly where we find the 101-bp insertion (position 874). The fragment we identified belongs to the sequence in position 285–419 of the Mrc1. Whether CRISPR/Cas9 favors insertion of a fragment of the retrotransposon close to the target site of our sgRNA or whether this is the result of an unrelated to CRISPR/Cas9 retrotransposon activity is an observation that requires further investigation. Interestingly, repair of CRISPR/Cas DSBs by the capture of DNA sequences deriving from retrotransposons has already been observed in animal cells [33], but not in algae.

We then wanted to investigate whether disruption of the *APT* gene had an effect on the growth of transformed cells. To do this, we set up liquid cultures for a representative CRISPR/Cas9 *APT*-disrupted 2-FA resistant line (obtained with glass beads), in TAP media, with and without the addition of 2-FA, and for a wild-type strain, also in TAP media with and without 2-FA. We observed that, as expected, the wild-type strain showed no proliferation in the presence of 2-FA, while there was no relevant difference in the growth of the wild-type strain growing in TAP medium and the transformed line growing in TAP media with and without 2-FA (Supplemental Figure S4). This result contrasts with what was observed in the mosses *Physcomitrella patens* and *Ceratodon purpureus*, where disruption of the *APT* gene resulted in impaired growth even when 2-FA resistant transformed lines were grown in the absence of this adenine analogue [34].

## 3. Discussion

Here we have shown that transient expression of Cas9 along a sgRNA targeted to the *APT* gene can be used for efficient gene disruption in the nuclear genome of *Chlamydomonas reinhardtii*. This is the first report in which Cas9 and a sgRNA have been shown to be effective for genome editing in *C. reinhardtii* without the need to carry out a positive selection of mutants resulting from the cointegration and expression of antibiotic resistance marker genes. In our strategy, we have shown that cells in which the *APT* gene is disrupted as a result of a brief transient expression of Cas9 and the sgRNA become insensitive to the otherwise toxic compound 2-FA, and thus can be easily and visually selected in selective media. Similar approaches have been carried out in the moss *Physcomitrella patens*, in which loss of function of the *APT* gene, also achieved with the use of the CRISPR/Cas9 system, has been shown to render the cells insensitive to 2-FA [34,35]. We obtained a gene disruption efficiency of 3 to 30% on preselected 2-FA-resistant colonies, a comparable and even slightly higher average efficiency with respect to the 9% reported by Greiner et al. (2017) when using paromomycin as a selective agent. It is important to point out that we have not used a promoter from *Chlamydomonas*, but rather the rice actin 1 promoter; nor have we used a *Chlamydomonas* codon-optimized Cas9 gene. It will be interesting to investigate whether there is an increase on the efficiency of edition when endogenous promoters are used and codon optimization is performed.

This is also the first report in which agitation with glass beads and the particle bombardment methods have been used for introduction of the plasmid-encoded Cas9 and sgRNA in *C. reinhardtii*. The former is a low-cost, widespread method for nuclear transformation in *C. reinhardtii* [25]. Meanwhile, particle bombardment is a more efficient method, albeit with the requirement of an expensive particle bombardment device, routinely used for chloroplast transformation in plants and algae. The particle bombardment method has in fact been used for CRISPR/Cas9 expression for genome editing in some plant species including rice [36], wheat [37] and maize [38]. The glass beads method involves agitation of cells with inexpensive glass beads in the presence of the transforming DNA with a vortex. This is a practical, yet efficient, method, and before this work had not been reported for CRISPR/Cas9-mediated edition of the nuclear genome in *C. reinhardtii*. This method has clear advantages, because of its relative simplicity and economy compared to the electroporation [39] and particle bombardment (which we have also reported here) methods. Our results indicate that the efficiency of gene disruption in preselected colonies seems to be 2-fold higher when transformation is carried out using the method of agitation with glass beads than when particle bombardment is used.

Use of the CRISPR/Cas9 technology was only recently developed to the point where it is sufficient for genome editing in *C. reinhardtii* [10,11]. Reports have relied mostly on the electroporation of preassembled ribonucleoprotein complexes [9,10,11,12], followed by selection of green to yellow phenotypically different colonies [9,12], or by selecting for colonies in which the selectable antibiotic resistance marker gene integrates in the genome. This possesses two disadvantages: the first one is that preassembled complexes have to be prepared at a cost that might make the technology inaccessible to laboratories with limited budgets (once assembled the RNP complexes have to be electroporated with the use of a mid to high cost equipment). Furthermore, while fast degradation and elimination of the RNP have been associated with a low off-target effect, they could also mean low edition efficiency in large genomes [40]. The second disadvantage is that there is always a high number of false positives, especially when using selectable genes (e.g., Photosynthetic genes) [10]. Furthermore, integration of the selectable marker is not always necessary; once the edition has been performed, there is no need for constant expression of Cas9 or the sgRNA. Here we have demonstrated that, thanks to an efficient reporter gene, transgene-free genome editing using plasmids is possible in *C. reinhardtii* and can potentially be used for CRISPR-Cas9-mediated disruption of genes of interest by combining sgRNAs targeting the APT gene and the gene of interest. Moreover, we have shown that the *C. reinhardtii APT* gene is a good reporter gene, without noticeable negative effect in the growth of transformed cells when it is disrupted, which could be used to improve the efficiency of gene editing through CRISPR-Cas9 in this organism.

As others have noted, with scientists from so many disciplines using the CRISPR technology, there does not seem to be a sign of slowing down in the creative applications pouring from it [41]. The development of tools for the use of CRISPR/Cas9 or other engineered and evolved nucleases in bacteria, yeast and mammalian cells is occurring at such an accelerated pace that scientists working with *Chlamydomonas* can hardly keep up; however, the recent progress in the field has been very encouraging, and one cannot but expect a rapid expansion on routine application of the technology in this and other marine [42,43] or photosynthetic organisms [44] for basic and applied science. The increasing application of these tools will improve the way we currently design and engineer biochemical pathways and gene circuits, and study gene function.

## 4. Materials and Methods

### 4.1. Cell Cultures

*Chlamydomonas reinhardtii* wild-type strain CC-125 (mt+) was obtained from the *Chlamydomonas* resource center (University of Minnesota, Minneapolis and Saint Paul, MN, USA). Wild-type and genome-edited strains were grown on standard Tris-Acetate-Phosphate (TAP) media [45] under the following conditions: 4000 lux of white LED light, 16 h light/8 h dark photoperiod at 25 °C. For solid cultures agar was used at 1.5% (*w*/*v*). For minimum inhibitory concentration assays, solid media was supplemented with 2-fluoroadenine (2-FA; Sigma-Aldrich, St. Louis, MO, USA) at a final concentration of 25, 50, 75, and 100 μg/mL depending on the required concentration.

### 4.2. RNA Guides and Cas9 Plasmids

For Cas9 protein expression, pAct-Cas9 plasmid was used; this vector was previously reported by [35] and contains a rice actin 1 promoter and a not optimised version of Cas9 from *Streptococcus pyogenes*. The SV40 nuclear localization signal sequence is placed in the 3’-end of the Cas9 coding sequence. For sgRNAs expression, the cassettes including the U6 promoter from *Chlamydomonas reinhardtii* [46], the target sequence, and the tracrRNA flanked by attB1 and attB2 Gateway^®^ sequences were synthesized as gBlock gene fragments (Integrated DNA technologies, Coralville, IA, USA). The cassettes were cloned into pDONR207 (Life Technologies, Carlsbad, CA, USA) by Gateway recombination to give the pEnt-sgRNA1/g01 and pEnt-sgRNA2/g02 plasmids. The target loci of sgRNA1 and sgRNA2 correspond to regions in exon 1 and exon 3, respectively, of the adenine phosphoribosyl transferase gene (Gene ID: 5717232).

### 4.3. Chlamydomonas reinhardtii Nuclear Transformation with Glass Beads

Nuclear transformation was carried out following the protocol described by Kindle, K.L. [25]. Briefly, 300 mg of 0.1 mm diameter glass beads (Thermo Fisher, Waltham, MA, USA) were added to a 1.5 mL tube. A total of 3 μg of each plasmid (pAct-CAs9, pEnt-sgRNA1, pEnt-sgRNA2) and 300 μL of *C. reinhardtii* cells (at a density of 0.5 × 10^8^, 1.5 × 10^8^ and 2.0 × 10^8^) were also added to the tube. The tube was vortexed at maximum speed for 35 s using a Daigger Vortex Genie 2 and the cells immediately transferred to a tube with 7 mL of liquid TAP and incubated at 25 °C for 24 h and with agitation at 250 rpm. The cell pellet was recovered by centrifugation at 5000× *g* for 5 min and plated in solid TAP medium supplemented with 2-fluoroadenine. Single 2-FA resistant colonies were plated again onto solid TAP media (100 μg/mL 2-FA) and incubated with a photoperiod as described above. For liquid cultures, TAP media was supplemented with 2-FA at 100 μg/mL.

### 4.4. Chlamydomonas reinhardtii Nuclear Transformation with Particle Bombardment

*Chlamydomonas* transformation was carried out by particle bombardment using conditions described by [47]. Briefly, wild-type *C. reinhardtii* cells grown in liquid TAP media were collected on mid-log phase by centrifugation at 3000× *g* for 5 min at room temperature. Cells were washed twice with sterile water, resuspended in fresh TAP media to reach a concentration of approximately 1 × 10^8^ cells, plated over solid TAP media supplemented with 2-FA (100 μg/mL) and placed under sterile conditions in a dark room for 2 h to dry the excess of liquid medium prior to bombardment. For particle preparation, 5 μL (1 μg/μL) of the nuclear transformation vector pAct-CAs9, and 5 μL (1 μg/μL) of the sgRNA vectors (pEnt-sgRNA1, pEnt-sgRNA2 or both), were added and mixed with 30 μL (30 mg/mL) of M-10 (0.7 µm) tungsten microparticles (Bio Rad, Hercules, CA, USA) in the presence of 20 µL of 2.5 mM spermidine and 50 µL of 0.1 mM CaCl_2_. Plated cells were subjected to particle bombardment with the Biolistic PDS-1000/He Particle Delivery System (Bio Rad) at 9 cm of distance using 1100 psi rupture disks (Bio Rad) and 20 mm Hg vacuum pressure within the chamber. After bombardment, plates were maintained in the dark for 24 h before being transferred to an incubation chamber at 25 °C with a 16:8 h light:dark photoperiod for 4–6 weeks. Resistant colonies were picked and transferred to freshly prepared solid TAP plates supplemented with 2-FA (100 μg/mL) and sub cultivated for 1–2 weeks.

### 4.5. PCR Amplification of APT Gene

Wild-type and 2-FA-resistant colonies were analyzed by colony PCR with Chelex-100 resin (Bio-Rad) following a previously reported method [47]. Briefly, a fraction of the colony was added to a 200 µL tube which contained 50 µL of a 5% Chelex-100 solution. Tubes were vigorously agitated and then briefly centrifuged and incubated for 20 min at 98 °C. One microliter of this solution was used per 50 µL PCR reaction. To amplify the 1132-bp PCR product, which contains the target site for both sgRNAS, primers APT-Fw (5′-CTTATTCACAAGGTCGAATC-3′) and NVDF278 (5′-TTGTGACGTTACACACTGCCTC-3′) were used (Supplementary Figure S1). PCR was carried out using KOD Hot Start DNA polymerase (Merck Millipore, Burlington, MA, USA) following the manufacturer’s instructions and using the following cycling conditions: 3 min at 94 °C, then 30 cycles of 30 s at 94 °C, 30 s at 60 °C, and 1 min at 72 °C, followed by a final extension of 5 min at 72 °C. The PCR products were analyzed by agarose gel electrophoresis (1%) stained with Gel Red (Biotium Inc., Hayward, CA, USA), bands around 1 kb were excised and purified with Wizard SV Gel and PCR Clean-Up System (Promega, Fitchburg, WI, USA).

### 4.6. Sequence Analysis

Sanger sequencing reactions of PCR products were carried out by Macrogen, Inc (Seoul, Korea). DNA sequences were assembled and analyzed with the software CodonCode Aligner v 6.0.2 (CodonCode Corporation v.v 6.0.2, Centerville, MA, USA).

## 5. Conclusions

In the present study, we have shown that transient expression of the plasmid-encoded CRISPR/Cas9 system can be efficiently used for targeting of the *APT* nuclear gene. We have demonstrated that the disruption efficiency can be as high as 30% when cells are agitated with glass beads for transformation and as high as 16% when cells are bombarded with tungsten particles. Targeted disruption of the *APT* gene, which yields lines tolerant to 2-fluoroadenine, to select transformed cells has been used in other organisms, but to the best of our knowledge, this is the first report in which it has been used for *C. reinhardtii* genome editing using the CRISPR/Cas9 technology.

## Supplementary Materials

Supplementary materials can be found at https://www.mdpi.com/1422-0067/20/5/1247/s1.
