# Supplementary material for: Efficient Editing of the Nuclear APT Reporter Gene in Chlamydomonas reinhardtii via Expression of a CRISPR-Cas9 Module
Source: Int J Mol Sci. 2019 Mar 12;20(5):1247. doi: 10.3390/ijms20051247 (PMC6429146; doi:10.3390/ijms20051247)
Supplement: Supplementary file 1 [file ijms-20-01247-s001.zip › ijms-429196 sp for final/Supplementary Fig 1.pdf]

Chlamydomonas reinhardtii- Adenine phosphoribosyltransferase gene(*apt* gene) Gene ID: 5717232

CTTATTCACAAGGTCGAATCAAACGCATTTTGTTTCAGTCTCTCTGAGTCTCGCGCCGCCCGCAACTTAC
APT-FW
AACCTTTCAGCACAAGCAACGCAAAGATGGCTGACGTTGAGGCCAAGAAGGAGATTATCAAGAATGCCAT
TCGTGGCATTCCTGACTTTCCCCACAAGGGTATTCTGTTCTGGGATGTCACCACCATCATGCTGAACCAC
CAGGCGTTCCAGTACAGCATTGACCTGTTCGCTGAGCAGTACAA**GGACAAGAAGATTGACGTGGTGG**CGG
GTAGGTGCAAGGGCAATGGAAGCAAGCATCGTGGGAACGCCGAAGCTATGCTGGGGTGGGGCAGGTAGAC
TGGCACCCCGCCCCGCCGGCGCCGTTATCGTCGCTTGCACCGTGCTTTCACCAACACCCCCCCCCGCGCG
CCCGCGCAGGCTTTGAGGCTCGTGGCCTCATCTTTGGCGCTCCTCTGGCTCTTGCGCTTGGGGTAGCCTT
TGTGCCGCTGCGCAAGCCCGGCAAGCTGCCCGGTACGTCAGCTGGCGTGCGGTTATGTTATGCGATCATG
TAGTGAGGGAGCTTGGCACGGCCGCCAACCGGGATCCGTGTAGCAGAGATATGGCGACCCCCGGCCGGTT
AAACCGCTGCACTGCGAGGGCCGCGGGCCGACCCTGTTGGCAATGAGGGGCGGCGGCCTGGCCGCCAGGG
CTGGTGCCCGGCGGTAACCGGTGGAGGGTCGTTGATGCGCGCACTTTGGTGACTCTTAAGGTGCAAACAG
AAGCTGTAGTTGGCAGCTTTCAGGTATCCTAACGCCTTTGGTCATGGGATTCATGCAGGTGACAAGATCA
GCGAGGAGTACAAGACCGAGTACAGCACGGACAAGATTGAGATGCACGTGGGCGCCAT**CCAGCCCGGGCA**
ACGCGTTGTGCTGGTAAGCGGGCCTGGTAGTGCACGCGGACGCAATGCTATGCAGGCTGTCAGGAGATGG
ACATGAGATGCTCGCCTTTTTGTGAAGACGGCAGCATGAGCAACCAAACAGTGATAGCTGTGAGACTGTC
ATCCGCTGTAGATCAGTTTGTCCGGGGCTTCAGAGGATGATTGTTTGTCACCGCATTGATTGTGACGTTA
NVDF 278
CACACTGCCTCCGTCATCCAGTACATGACCGCTTCCCAACACCTCAAACGCATCAGTAAACCCTGCGGCG
CAGTAGCACATCGTGCATCGCGCCATGGATAGACTAATGCCGGCCCCTTCATGTTTGAACCCATTGCAGG
TTGATGACCTGATTGCCACCGGCGGCACCCTTGCCGCCGGCATCAACCTCGTCAGTAAGTCTCACATGCG
CCTTACACACACGGTGCAGTGCACTTTCTTCCATCTTCAATGGTGGTCTGCGCCATGAACGCAAGCAATG
CCACTACCCTTGCCTTTACATGGAATCCAGCCACTTCCTGCCATCATACCCCTCACCTACCGGCACTTCT
CCAGTACCACTTCAACTCATTGCAAACTTAGCCACGCACCCCAAGGCCGCTCACCACGAATCCATTCGCC
CTCACCGCCCCACAGAGAAGGCCGGCGGTGTGGTGGTGGAGGCGGCGTGCGTCATCGAGCTGCCCTTCCT
CAAGGGCCGCGACAAGATCCAGGGCACCGACCTCTTCGTGCTCGTGGAGAAGGAGGGCCTGTAAGCAACT
CCACTGCCAAGACCGCAGCCGTGCATATCTAGCTAGTGCACCCGGCAGCCGTATGAAAGCATTCTCGGTC
TGTGCACCGTCGCACATTATGCAGCCGCAACAGGCCTTGGATCGACCACGGCACGTCGAGCCGAAGTGCC
CGACCTAGTACACTTGACCACGGTTGATTGCTTTGGAAGTCTGTGATCGCAACCTTAAACCCGATAGTAC
ATTGGCCTGGGCCACGGCGCCACCCCGGCGCAGCGCGTGAGCCTGACTGGTTAGGTGAAGCCCCTCCGGG
AGCTCATTACCCCGGGGGCACCGGTGCCGTGACTACTGTTGCCGGTATGGGCTGGCACGTTTAAGTGCGT
GCGCCATTGAGCTCACGAACTGGTCCTTTGTGAGGTTGCTCCCGGGCTTGACGCCCACCTCCGCAAGGTC
CGCTGCTGGCCCCAAGATGACTTGAAGCGCACGGTTTGCCTGCTCTGCCGTGACCGTGCCGCGTTTTGTA
ATGTCGAACATGCCAAACATTGTTTCCAAGTCCTGCTCGTTGATGAGGGGTGCTGTCCCCGCGACCTTCA
CATTCTCAAGGTAC
