# Supplementary material for: Efficient Editing of the Nuclear APT Reporter Gene in Chlamydomonas reinhardtii via Expression of a CRISPR-Cas9 Module
Source: Int J Mol Sci. 2019 Mar 12;20(5):1247. doi: 10.3390/ijms20051247 (PMC6429146; doi:10.3390/ijms20051247)
Supplement: Supplementary file 1 [file ijms-20-01247-s001.zip › ijms-429196 sp for final/Supplementary material figure legends.docx]

Figure S1. *Chlamydomonas reinhardtii* Adenine phosphoribosyltransferase gene (apt gene). Sequence of the *APT* gen was obtained from https://www.ncbi.nlm.nih.gov/ under Gene ID: 5717232. APT gene has 5 exons which are highlighted with light grey color, sequence of sgRNA-1 (Exon 1) and sgRNA-2 (Exon 3) are in bold font and primers APTFW and NVDF278 are underlined with bold arrows.

Figure S2. Gel electrophoresis of APT gene PCR amplification. PCR products obtained with primers APTFW and NVDF278 from resistant colonies transformed with (from top to bottom) SgRNA1; SgRNA2; and both SgRNA1 – SgRNA2.

Figure S3. Secondary structure prediction of single guide RNA. Sequences of SgRNA-1 and SgRNA-2 were introduced in RNAfold web server (<http://rna.tbi.univie.ac.at/cgi-bin/RNAWebSuite/RNAfold.cgi>) for secondary structure view. sgRNA-1 ggacaagaagattgacgtgg GTTTTAGAGCTAGAAATAGCAAGTTAAAATAAGGCTAGTCCGTTAT
CAACTTGAAAAAGTGGCACCGAGTCGGTGC and sgRNA-2 gcacaacgcgttgcccgggcGTTTTAGAGCTAGAA
ATAGCAAGTTAAAATAAGGCTAGTCCGTTATCAACTTGAAAAAGTGGCACCGAGTCGGTGC.

Figure S4. Growth curves of *C. reinhardtii* wild-type and transformed 2-FA resistant lines in TAP media with and without 2-FA. Supplemental Data 1. Raw data from sequencing results.
