# Supplementary figures and images for: Efficient Editing of the Nuclear APT Reporter Gene in Chlamydomonas reinhardtii via Expression of a CRISPR-Cas9 Module
Source: Int J Mol Sci. 2019 Mar 12;20(5):1247. doi: 10.3390/ijms20051247 (PMC6429146; doi:10.3390/ijms20051247)

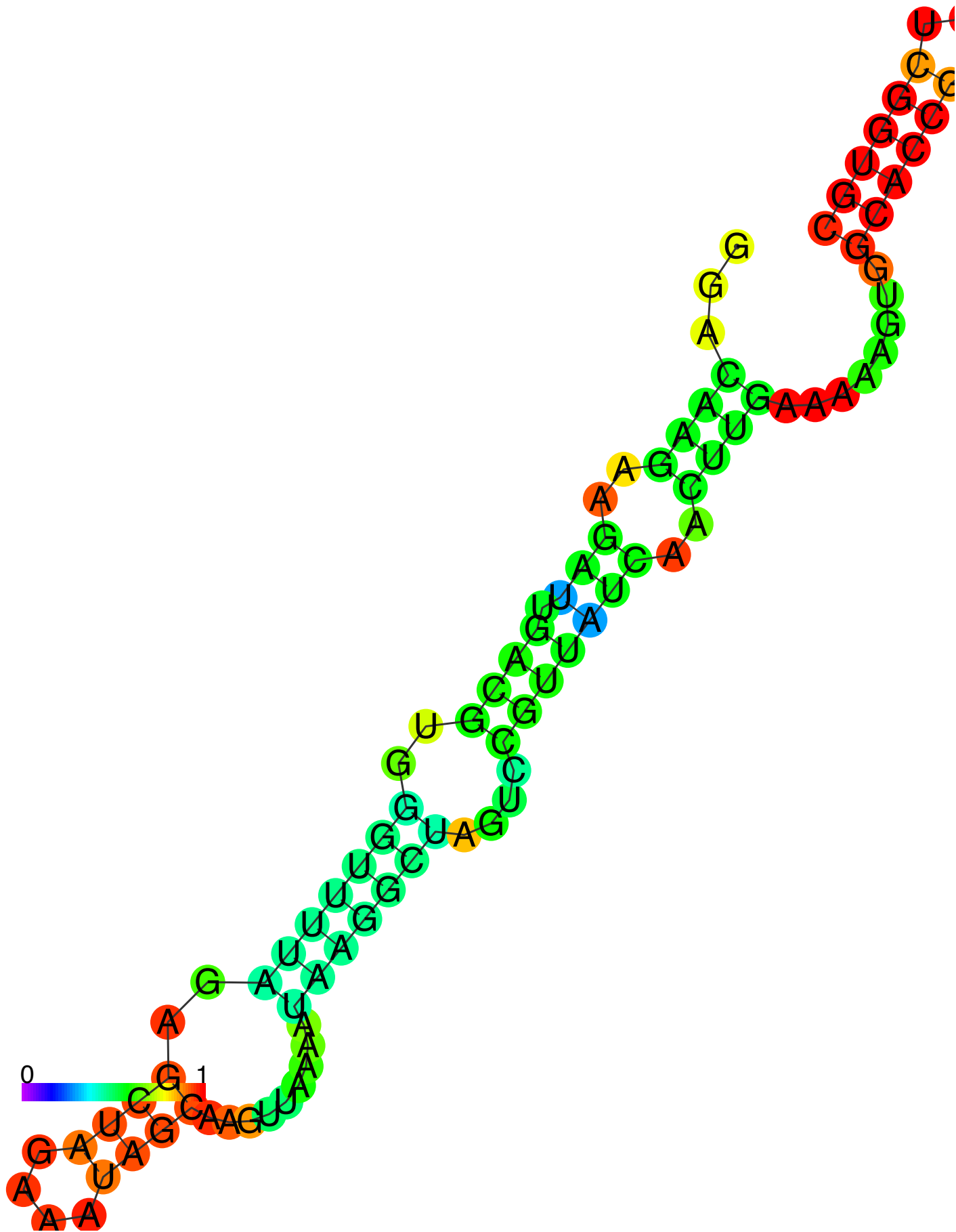

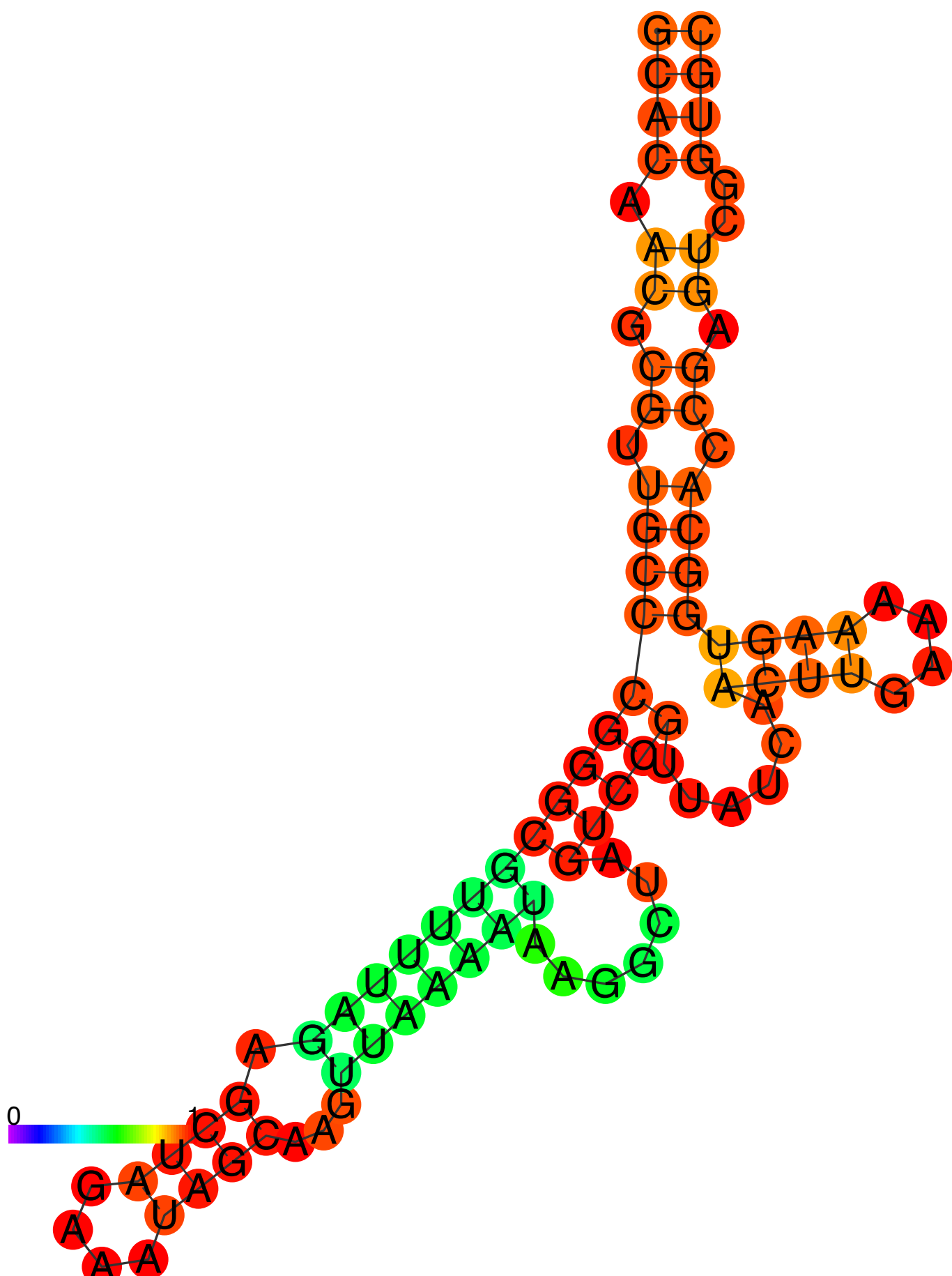

Supplement: Supplementary file 1 [file ijms-20-01247-s001.zip › ijms-429196 sp for final/Supplemantary Fig 3.pdf]

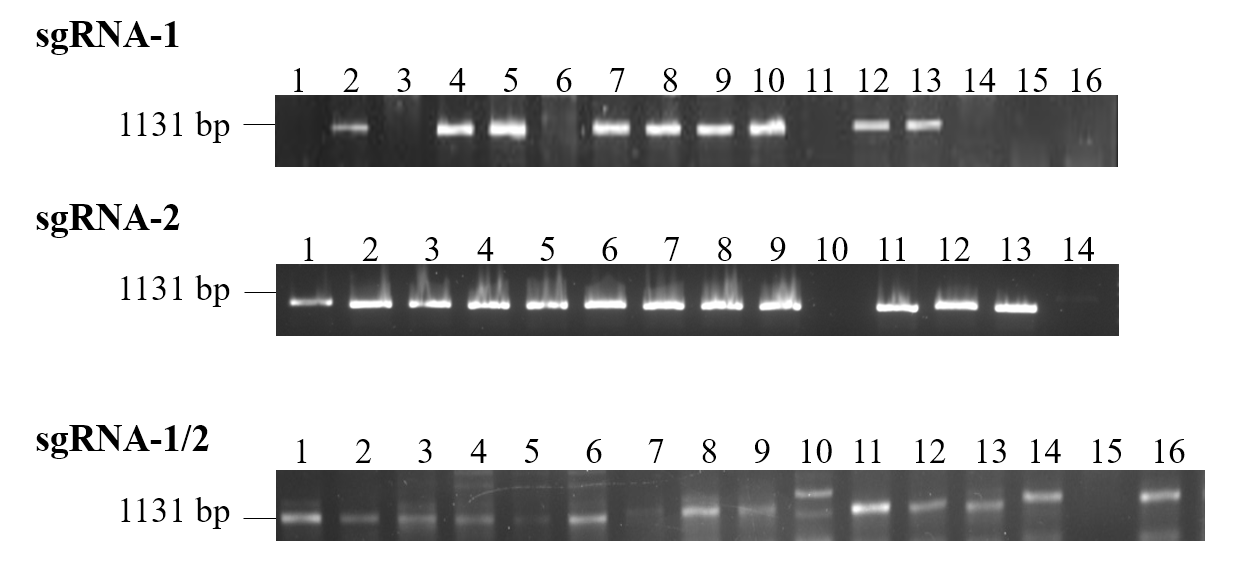

Supplement: Supplementary file 1 [file ijms-20-01247-s001.zip › ijms-429196 sp for final/Supplementary Fig 2.tif]

# Wild-type and transformed *Chlamydomonas reinhardtii* lines with and without 2-FA

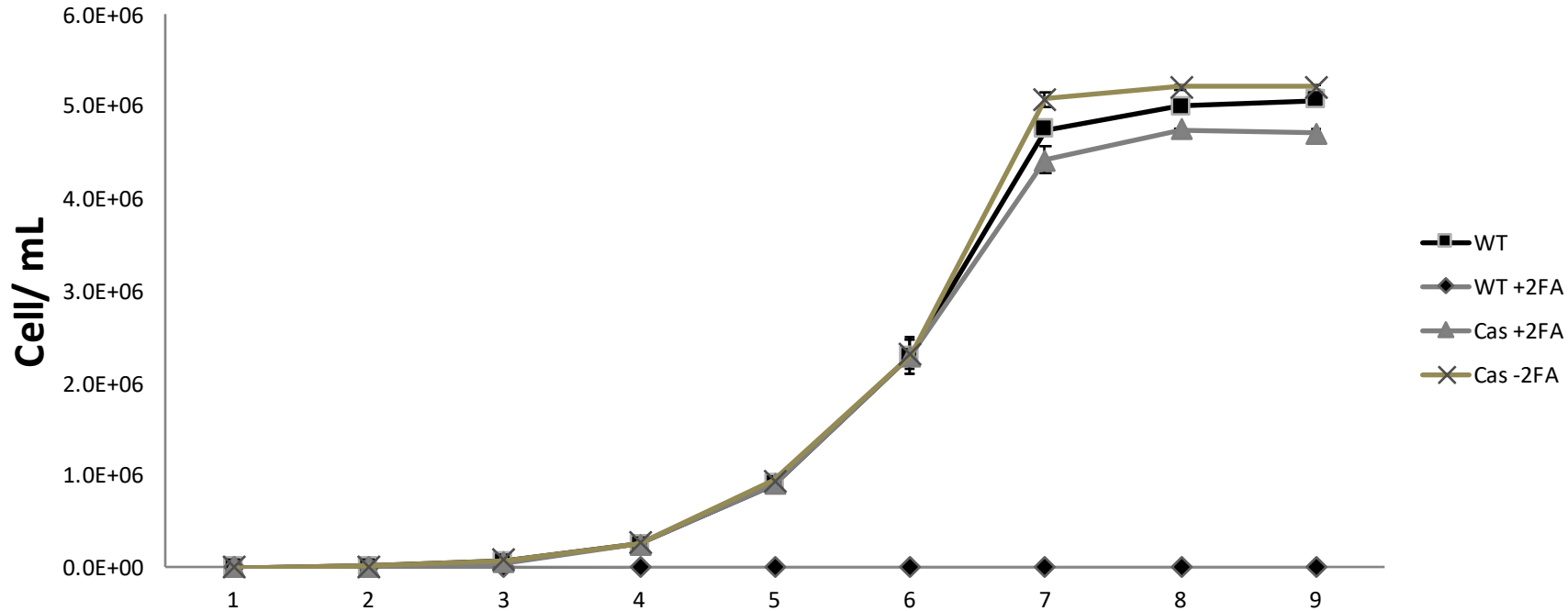

Supplement: Supplementary file 1 [file ijms-20-01247-s001.zip › ijms-429196 sp for final/Supplementary Fig 4.pdf]
